# Disruption of androgen signaling during puberty affects Notch pathway in rat seminiferous epithelium

**DOI:** 10.1186/s12958-020-00582-3

**Published:** 2020-04-16

**Authors:** Alicja Kamińska, Sylwia Marek, Laura Pardyak, Małgorzata Brzoskwinia, Piotr Pawlicki, Barbara Bilińska, Anna Hejmej

**Affiliations:** Department of Endocrinology, Faculty of Biology, Institute of Zoology & Biomedical Research, Jagiellonian University, Gronostajowa 9, 30-387 Krakow, Poland

**Keywords:** Notch signaling, Androgens, Testis, Puberty

## Abstract

**Background:**

Onset of spermatogenesis at puberty is critically dependent on the activity of hypothalamic-pituitary-gonadal axis and testosterone production by Leydig cells. The aim of this study was to examine whether activation of Notch receptors and expression of Notch ligands and effector genes in rat seminiferous epithelium are controlled by androgen signaling during puberty.

**Methods:**

Peripubertal (5-week-old) Wistar rats received injections of flutamide (50 mg/kg bw) daily for 7 days to reduce androgen receptor (AR) signaling or a single injection of ethanedimethane sulphonate (EDS; 75 mg/kg bw) to reduce testosterone production. Gene and protein expressions were analyzed by real-time RT-PCR and western blotting, respectively, protein distribution by immunohistochemistry, and steroid hormone concentrations by enzyme-linked immunosorbent assay. Statistical analyses were performed using one-way ANOVA followed by Tukey’s post hoc test or by Kruskal-Wallis test, followed by Dunn’s test.

**Results:**

In both experimental models changes of a similar nature in the expression of Notch pathway components were found. Androgen deprivation caused the reduction of mRNA and protein expression of DLL4 ligand, activated forms of Notch1 and Notch2 receptors and HES1 and HEY1 effector genes (*p* < 0.05, *p* < 0.01, *p* < 0.001). In contrast, DLL1, JAG1 and HES5 expressions increased in seminiferous epithelium of both flutamide and EDS-treated rats (*p* < 0.05, *p* < 0.01, *p* < 0.001).

**Conclusions:**

Androgens and androgen receptor signaling may be considered as factors regulating Notch pathway activity and the expression of *Hes* and *Hey* genes in rat seminiferous epithelium during pubertal development. Further studies should focus on functional significance of androgen-Notch signaling cross-talk in the initiation and maintenance of spermatogenesis.

## Background

Spermatogenesis is a process in which germ cells undergo precisely timed sequence of phases of proliferation and differentiation to produce male gametes, spermatozoa. Onset of spermatozoa production at puberty is critically dependent on the activity of hypothalamic-pituitary-gonadal axis and testosterone production by Leydig cells [1]. Testosterone acts to regulate spermatogenesis predominantly through androgen receptor (AR), a member of nuclear receptor subfamily 3. The majority of evidence indicates that differentiating germ cells do not express the AR and Sertoli cells are considered as major mediators of androgen action in the control of spermatogenesis [2]. In rat Sertoli cells the AR is detected from postnatal day (PD) 5. The number of AR expressing Sertoli cells and condensation of AR nuclear staining gradually increase with age and stage-dependent expression pattern appears between PD 21 and 35, preceding an increase of circulating testosterone from PD 35 [3–5]. Studies on Sertoli cell-specific AR knockouts or gain-of-function transgenic mouse models clearly demonstrated the connection between androgen-induced Sertoli cell maturation and germ cell progression through meiosis [6, 7]. At puberty onset androgens drive Sertoli cell maturation, repressing antyműllerian hormone (AMH) expression and controlling the formation of blood-testis barrier. As a consequence, a supportive environment for the completion of germ cell meiosis is created and the full progression of spermatogenesis occurs. Maintenance and dynamics of the blood-testis barrier, as well as Sertoli cell–spermatid adhesion and sperm release from seminiferous epithelium (spermiation) are also dependent on androgen action in Sertoli cells [1].

Although numerous studies have been performed to elucidate the role of androgens in initiation and maintenance of spermatogenesis, the molecular pathways that link androgen signaling to the control of germ cell development are still not completely understood. Herein, we hypothesize that Notch signaling pathway may be regulated by androgen action in seminiferous epithelium at puberty. Notch pathway is an evolutionarily conserved juxtacrine signaling network which plays a role in coordinating cell differentiation and cell-fate decisions in many organs. Activation of this pathway is induced by binding of the extracellular domain of Notch receptors with membranous ligands present on neighboring cells. This results in the cleavage of the Notch extracellular domain by disintegrin and metalloproteinases (ADAMs), while intracellular domain is cleaved by the γ-secretase complex. Released Notch intracellular domain (NICD) translocates to the nucleus, where it builds up coactivator complex composed of recombination signal binding protein RBP-J, Mastermind-like 1 (MAML1) and NICD, together with histone acetylases [for review see [8]. The Notch coactivator complex facilitates histone acetylation leading to the expression of target genes. Several Notch effector genes belonging to hairy/enhancer of split (*Hes*) and Hes-related with YRPW motif (*Hey*) families are expressed in the testis [9]. HES and HEY proteins are known as transcriptional repressors that act directly on target promoters to regulate cellular processes [10].

In the control of spermatogenesis special attention was put to Notch1 receptor signaling in Sertoli and germ cells. Importance of Notch1 signaling was evidenced by studies on gain-of-function models. Conditional activation of the Notch1 intracellular domain (N1ICD) in germ cells caused increase of germ cell apoptosis, reduction in sperm counts and a progressive loss of testis weight with age. On the other hand, constitutive activation of Notch1 signaling in Sertoli cells led to premature differentiation of gonocytes, followed by gonocyte apoptosis, which resulted in infertility [11]. Although Notch1 knockout in either Sertoli or germ cells had no effect on spermatogenesis, likely due to redundancy of Notch receptors, pharmacological inhibition of Notch pathway caused spermatogenesis defects [11–13].

During postnatal development of rodent testis changes in the pattern of Notch pathway components expression were observed; specifically Notch1 receptor replaces Notch3 in Sertoli cells of peripubertal mouse [9, 14, 15]. The role and regulation of Notch2 was demonstrated to date in fetal mouse testis [16] and in tumor Leydig cells [17], but only scarce data exist on its localization in seminiferous epithelium [18, 19].

It was previously reported that circulating levels of testosterone directly affected Notch signaling in progenitor Leydig cells, regulating the maintenance of fetal Leydig cell population [20], but the potential function of androgens in the control of Notch pathway activity in seminiferous epithelium has not been explored yet. Given that androgens play a prominent role in the initiation of sperm production around puberty, the involvement of Notch pathway in the androgen responses of testicular cells is intriguing. Therefore, in the present study we focused on the Notch receptors activation and expression of Notch ligands and effector genes in rat seminiferous epithelium following manipulations of androgen signaling or production during puberty. Since some components of Notch pathway, as well as the AR are also present in the Leydig cells, these cells were included into analyses where needed.

## Methods

### Animals and treatments

Pubertal (5-week-old) Wistar rats (*Rattus norvegicus*) were obtained from the animal facility at Faculty of Pharmacy, Jagiellonian University, Krakow. The rats were maintained on a 12-h light and 12-h dark cycle and were allowed food and water *ad libitum*. Rats (*n* = 6/each group) received sc. injections of flutamide (50 mg/kg bw) daily for 7 days as described previously [21] or single injection of ethanedimethane sulphonate (EDS; 75 mg/kg bw) as described previously [22, 23]. Flutamide reduces androgen action by inhibiting AR transcriptional activity, whereas EDS selectively destroys Leydig cells, the main source of androgens, leading to significant decrease in testosterone production [24, 25]. Vehicle-treated as well as saline-treated controls were performed (*n* = 6 each group). Since there were no differences neither in testosterone levels nor in the expression of studied genes (data not shown) between vehicle-treated as well as saline-treated groups, only the latter was presented in the Results section as a control group.

Animals were sacrificed by inhalation of 4–5% (v/v) isoflurane at day 8 after first flutamide or EDS injection. Testes were dissected immediately and were either frozen (one testis from each animal, *n* = 6/each group) in liquid nitrogen for real-time RT-PCR and western blot analyses or fixed either in Bouin’s fluid (Sigma-Aldrich; *n* = 3/each group) or 4% paraformaldehyde (Santa Cruz Biotechnology; *n* = 3/each group). Paraffin tissue sections were used for eosin-hematoxylin staining and immunohistochemistry. Blood was collected and testosterone concentrations were measured in blood plasma using DRG Testosterone ELISA (DRG International) according to the manufacturer’s instructions. All animal procedures followed the guidelines of the Polish Animal Welfare act and were approved by the 2nd Local Institutional Animal Care and Use Committee in Krakow, Poland (189/2018 and 85/2019).

### RNA isolation, reverse transcription and real-time quantitative RT-PCR

Total RNA was extracted from the testes (*n* = 6/each group) with TRIzol® reagent (Life Technologies) according to the manufacturer’s instructions. Contaminating DNA products were removed with TURBO DNA-free™ Kit (Ambion) according to the manufacturer’s instructions. The quality and yield of the RNA were evaluated by measuring the A260:A280 ratio (NanoDrop ND2000 Spectrophotometer, Thermo Scientific) and by electrophoresis. A260:280 ratios not lower than 1.9 were accepted for cDNA synthesis.

cDNA was synthetized using purified RNA and the High-Capacity cDNA Reverse Transcription Kit (Applied Biosystems) according to the manufacturer’s instructions. Parallel reactions for each RNA sample were run in the absence of reverse transcriptase to evaluate genomic DNA contamination.

StepOne Real-time PCR system (Applied Biosystems) was used for real-time RT-PCR analyses with the cDNA templates as described above and primers listed in Table 1 (Institute of Biochemistry and Biophysics, Polish Academy of Science). Amplification efficiency was determined as described by Svec et al. [26]. All PCR assays displayed efficiency between 95 and 102%.

**Table 1:** Primers used in real-time RT–PCR analyses

Genes	Forward sequence	Reverse sequence	Product size (bp)	Annealing temp.(°C)
*Actb*	AAGTACCCCATTGAACACGG	ATCACAATGCCAGTGGTACG	257	52
*B2m*	GGACTGGTCTTTCTATATCCTGGC	GATCACATGTCTCGATCCCAGTAG	150	58
*Cldn11*	TGTCAACAGCAGCAAGATGGCG	GCTCCAAGGGCCTGTGGGC	332	57
*Dll1*	TCAGATAACCCTGACGGAGGC	AGGTAAGAGTTGCCGAGGTCC	185	56
*Dll4*	GCTGGAAGTGGATTGTGG	CTTGTCGCTGTGAGGATAC	405	51
*Hes1*	GGCAGGCGCACCCCGCCTTG	GCAGCCAGGCTGGAGAGGCT	170	62
*Hes5*	ACCGCATCAACAGCAGCATT	AGGCTTTGCTGTGCTTCAGGT	135	55
*Hey1*	AAAGACGGAGAGGCATCATCG	GCAGTGTGCAGCATTTTCAGG	126	55
*Jag1*	AACTGGTACCGGTGCGAA	TGATGCAAGATCTCCCTGAAAC	216	54
*Notch1*	GCAGCCACAGAACTTACAAATCCAG	TAAATGCCTCTGGAATGTGGGTGAT	445	56
*Notch2*	GCTGTCCTCTTCATGCTGCA	AGCAGAAGTCAAGACAGTC	405	51
*PCI*	TGACCCCCAAAAGGACCAC	TGGTCCAGGTAGTAGGAATACCCA	237	54
*Rhox5*	TCATCATTGATCCTATTCAGGGTATG	CTCTCCAGCCTGGAAGAAAGC	380	55
*Rn18s*	CATTCGAACGTCTGCCCTAT	GTTTCTCAGGCTCCCTCTCC	128	56

Detection of amplification products of tested genes and the reference genes (*B2m*, *Rn18s* and *Actb*) was performed with 10 ng cDNA, 0.5 μM primers, and SYBR Green master mix (Applied Biosystems) according to the manufacturer’s instructions. To confirm amplification specificity, the PCR products were subjected to melting curve analysis and subsequent agarose gel electrophoresis. In all real-time RT-PCR reactions, a negative control corresponding to RT reaction without the reverse transcriptase enzyme and a blank sample were carried out (data not shown). mRNA expressions were normalized to the mean expression of the reference genes *Rn18s*, *B2m* and *Actb* mRNA (relative quantification, RQ = 1) with the use of the 2 ^− ΔΔCt^ method, as previously described [27].

### Western blot analysis

The proteins were extracted from testicular tissue (*n* = 6/each group) with a cold RIPA buffer (Thermo Scientific) supplemented with protease inhibitors (Sigma-Aldrich). Separation of protein preparations by SDS-PAGE under reducing conditions and transfer of proteins to polyvinylidene difluoride membranes were performed as described before [28]. Nonspecific binding sites were blocked with a solution of 5% (wt/v) non-fat dry milk containing 0.1% (v/v) Tween® 20. Next the membranes were incubated with the respective primary antibody (Table 2) at 4 °C overnight, followed by a horseradish peroxidase-conjugated secondary antibody (1:3000; Vector Laboratories) for 1 h at room temperature. Proteins were detected by chemiluminescence [29], and documented with a ChemiDocTM XRS+ System (Bio–Rad Laboratories). All immunoblots were stripped and reprobed with an anti-β-actin antibody as the loading control [30]. The molecular weights of target proteins were estimated by reference to standard proteins (Sigma–Aldrich). To obtain quantitative results, immunoblots were analyzed densitometrically using the ImageLab software (Bio–Rad Laboratories). Each data point was normalized against its corresponding actin data point.

**Table 2:** Details of primary antibodies used for western blot and immunohistochemistry

Antibody	Host species	Vendor	Cat. Number	Dilution
Anti-actin	Mouse	Sigma-Aldrich	A2228	1:3000 (WB)^a^
Anti-DLL1	Rabbit	Sigma-Aldrich	SAB2100593	1:1000 (WB), 1:100 (IHC)
Anti-DLL4	Rabbit	Abcam	AB7280	1:2000 (WB), 1:400 (IHC)
Anti-HES1	Rabbit	Thermo Fischer Scientific	PA5–28802	1:1000 (WB); 1:100 (IHC)
Anti-HES5	Goat	Santa Cruz Biotechnology	sc-13,859	1:300 (WB), 1:50 (IHC)
Anti-HEY1	Rabbit	Thermo Fischer Scientific	PA5–40553	1:500 (WB), 1:100 (IHC)
Anti-JAG1	Rabbit	Thermo Fischer Scientific	PA5–72843	1:3000(WB), 1:600 (IHC)
Anti-LHR	Rabbit	Santa Cruz Biotechnology	sc-25,828	1:1000 (WB), 1:200 (IHC)
Anti-N1ICD	Rabbit	Abcam	Ab8925	1:1000 (WB), 1:200 (IHC)
Anti-N2ICD	Rabbit	Sigma-Aldrich	SAB4502020	1:500 (WB), 1:200 (IHC)

^a^*WB* western blot, *IHC* immunohistochemistry

### Immunohistochemistry

Immunohistochemistry was performed on 5 μm sections of testicular tissue. Antigen retrieval, endogenous peroxidase neutralization and blocking of non-specific binding sites were performed as described previously [31]. Thereafter, the sections were incubated overnight at 4 °C with a primary antibody (Table 2). On the next day, a biotinylated goat anti-rabbit or horse anti-goat secondary antibody (1:400; Vector Laboratories) was applied for 60 min. The staining was developed with an avidin-biotinylated horseradish peroxidase complex solution (1:100; VECTASTAIN Elite ABC Reagent, Vector Laboratories) for 30 min, followed by 0.05% 3.3′-diaminobenzidine tetrachloride containing 0.01% (v/v) H_2_O_2_ and 0.07% (wt/v) imidazole. Sections were counterstained with Mayer’s hematoxylin. All slides within an experiment were processed identically at the same time so that the staining intensity among different sections of the testis could be compared. To validate specificity of primary antibodies used for immunohistochemistry, western blotting was performed (for detail see subsection "Western blot analysis"). Negative controls in the absence of primary antibodies were performed for each immunostaining. Sections were examined with a Nikon Eclipse Ni microscope (Nikon Instech Co., Ltd., Tokyo, Japan).

For semi-quantitative analysis of immunohistochemical reaction testicular sections were recorded using Nikon Eclipse Ni microscope (Nikon Instech Co., Ltd., Tokyo, Japan) equipped with × 100 objective lens (NA 1.4) and high-definition DS-Fi2 video camera (Nikon Instech Co., Ltd.). Approximately 40 images from testicular sections of each examined animal (*n* = 3/each group) were analyzed. The outlines of the cells were marked manually and the gray level (GL) of marked areas was measured. Protein levels in Sertoli and germ cells were measured throughout all stages of seminiferous epithelium with the public domain ImageJ software (National Institutes of Health, Bethesda, MD). The intensity of the reaction was expressed as relative optical density (ROD) of diaminobenzidine brown reaction products and was calculated with the following formula:

$$ \mathrm{ROD}={\mathrm{OD}}_{\mathrm{spicemen}}/{\mathrm{OD}}_{\mathrm{background}}=\log \left({\mathrm{GL}}_{\mathrm{blank}}/{\mathrm{GL}}_{\mathrm{specimen}}\right)/\log \left({\mathrm{GL}}_{\mathrm{blank}}/{\mathrm{GL}}_{\mathrm{specimen}}\right) $$

where OD is optical density, GL_specimen_ – the gray level for the stained area, GL_background_ – gray level of unstained area (background) and GL_blank_ – the gray level measured after the slide was removed from the light path [32]. Data were expressed as means ± *SD*.

### Morphometry

Hematoxyline and eosine (H-E) staining was performed on 5 μm sections of Bouin-fixed paraffin-embedded testicular tissue. The diameter of the seminiferous tubules was measured at × 100 magnification using ImageJ software. On average 60 circular tubules were measured per slide. When the tubular sections were slightly oval, only the smaller diameter was measured. Mean was determined for each animal from control and treatment groups. Data were expressed in μm as means *± SD*.

The area of the interstitium occupied by Leydig cells was calculated from ImageJ measurements of freehand outlines drawn along the circumference of LHR-positive cell clusters. The area of Leydig cells was determined at × 400 magnification in 40 random fields of vision for each section examined, and was expressed as a percentage of the area obtained from control group calculations *± SD*. For the control group, area was adopted as 100%.

Numbers of abnormal, retained or degenerating spermatids were counted on H-E sections and expressed as a percentage of total number (100%) of elongated spermatids counted. For each animal, elongated spermatids from 200 seminiferous tubules cross-sections were counted. Data were expressed as means ± *SD*.

### Statistical analysis

Statistical differences were assessed using one-way ANOVA followed by Tukey’s post hoc comparison test when the data showed a normal distribution and homogeneity of variances. Otherwise, a nonparametric Kruskal-Wallis test, followed by Dunn’s test were used when the data did not show a normal distribution and/or homogeneity of variances. Statistical analyses were performed on raw data using Statistica 10 software (StatSoft Inc.). Data from real-time RT-PCR and densitometry analyses were expressed in arbitrary units (AU) as means *±* SD. Data from testosterone assay were expressed in ng/mL as means *± SD*. Data were considered statistically significant at *p < 0.05, **p < 0.01, ***p < 0.001.

## Results

**Fig. 1:**
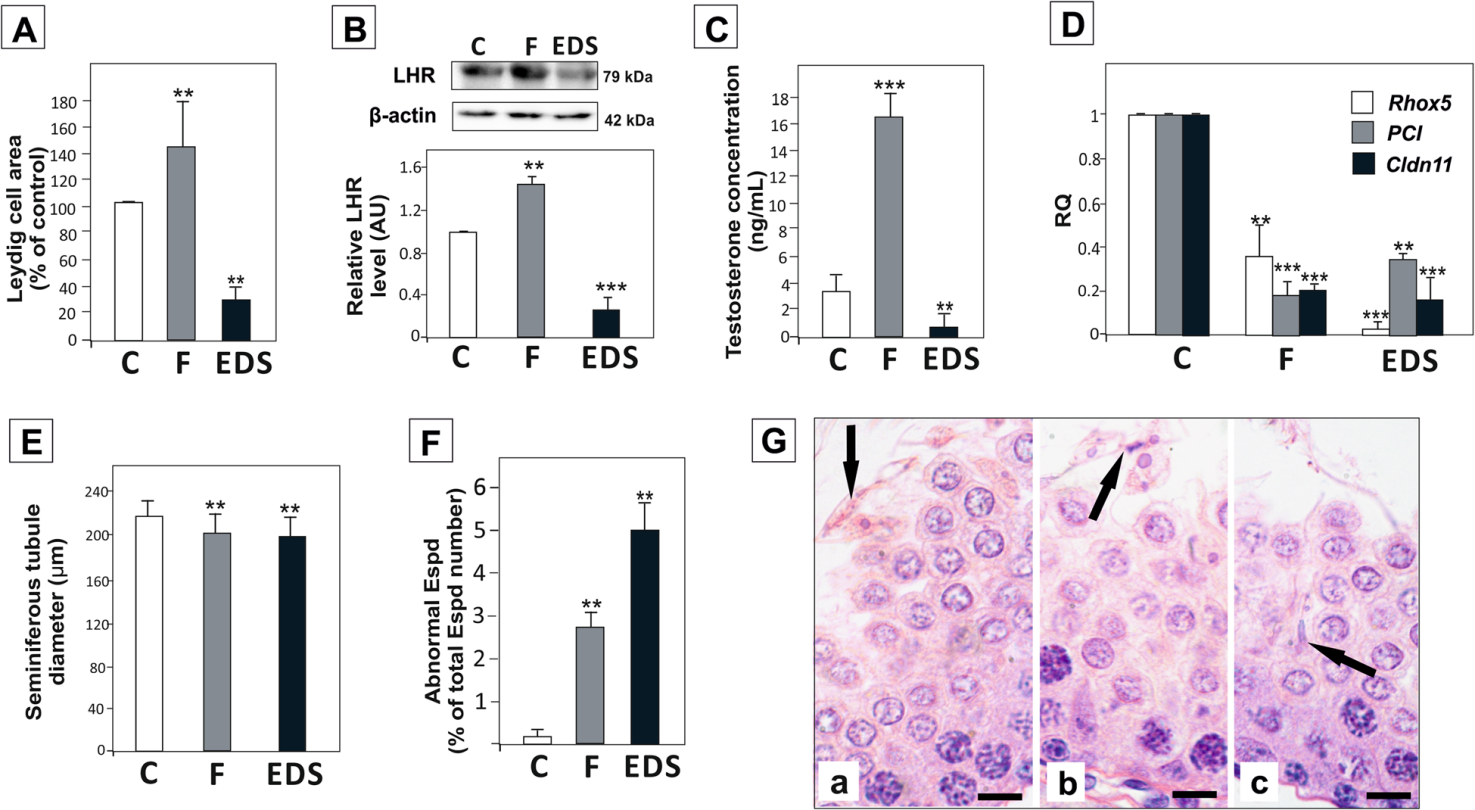
Effect of flutamide and EDS-treatment on testicular morphology, plasma testosterone concentration, and the expression of *Rhox5*, *PCI* and *Cldn11*. (A) Area occupied by Leydig cells, (B) LH receptor (LHR) expression in the testis, (C) plasma testosterone concentrations, (D) mRNA expression of *Rhox5*, *PCI* and *Cldn11*, (E) seminiferous tubule diameter, (F) percentage of abnormal or degenerating elongated spermatids (Espd) in seminiferous epithelium of control (C), flutamide (F) and EDS-treated (EDS) rats, and (G) representative images of seminiferous epithelium morphology, showing examples of spermatid degeneration (arrows) in flutamide (Ga) and EDS-treated (Gb, Gc) males; scale bar = 15 μm. For details see Material & methods. Significant differences from control values are denoted as *p < 0.05, **p < 0.01, and ***p < 0.001

Efficacy of androgen deprivation by flutamide or EDS was examined through analysis of luteinizing hormone receptor (LHR; marker of Leydig cells), plasma testosterone concentrations, and the expression of three genes regulated by androgens in Sertoli cells, *Rhox5*, *PCI* and *Cldn11* [33]. Blockade of the AR by flutamide inhibits classical testosterone signaling in testicular cells as well as the negative feedback of testosterone on the pituitary gland. This leads to an increase in circulating luteinizing hormone, resulting in stimulation of Leydig cell [34]. As expected, in the present study flutamide treatment led to Leydig cell hypertrophy and significant increase in testosterone secretion (*p* < 0.01) (Fig. 1A-C). EDS, an alkylating agent, which specifically eliminates Leydig cells, caused a 70% reduction of area occupied by Leydig cells within 1 week (*p* < 0.01) (Fig. 1A). This was correlated with marked reduction of LHR level in testicular homogenates (*p* < 0.001) (Fig. 1B). Plasma testosterone concentrations were also much lower than in control males (*p* < 0.01) (Fig. 1C). In agreement, we found that the expression of *Rhox5*, *PCI* and *Cldn11*, was significantly downregulated in the testes of both flutamide and EDS-treated males (*p* < 0.01, *p* < 0.001) (Fig. 1D). There was about 10% reduction of seminiferous tubules diameter in the experimental groups *vs*. control group (*p* < 0.01) (Fig. 1E), and abnormal or degenerating elongated spermatids were observed in seminiferous epithelium (Fig. 1F, G). However, all germ cell populations (spermatogonia, spermatocytes, round and elongated spermatids) were present in all groups of rats. Thus, short-term androgen signaling disruption models used herein allowed for efficient androgen signaling reduction without producing profound alterations in germ cell composition in seminiferous epithelium of peripubertal males.

**Fig. 2:**
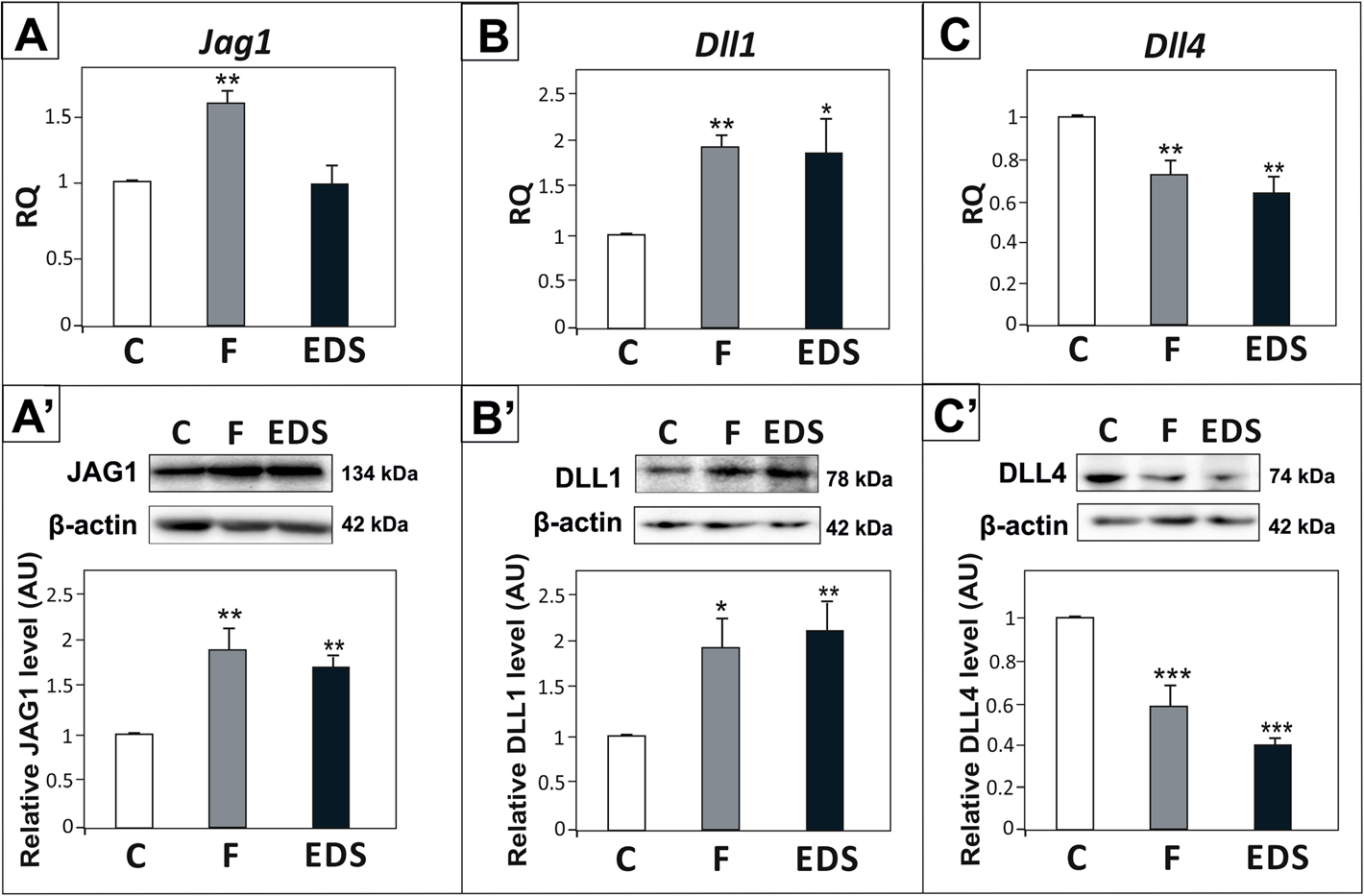
Effect of flutamide (F) and EDS on *Jag1*, *Dll1* and *Dll4* expression in rat testis. (A – C) Relative expression of *Jag1*, *Dll1* and *Dll4* mRNAs was determined using real-time RT-PCR analysis. The histograms are the quantitative representation of data of three independent analyses (*n* = 6 each group). The expression values of the individual genes were normalized to the mean expression of the reference genes (*Rn18s*, *B2m* and *Actb*) as an internal control. Relative quantification (RQ) is expressed as mean ± SD. Significant differences from control values are denoted as **p < 0.01 and ***p < 0.001. (A’ – C′) Relative protein expression of JAG1, DLL1 and DLL4 was determined using western blot. The histograms are the quantitative representation after densitometry of data (mean ± SD) of three independent analyses (*n* = 6 each group). The relative level of studied protein was normalized against its corresponding actin data point. The protein levels within the control group were arbitrarily set at 1. Significant differences from control values are denoted as *p < 0.05, **p < 0.01, and ***p < 0.001

Effects of androgen deprivation on the expression of JAG1, DLL1 and DLL4 ligands were differential. We have found that mRNA and protein expression of DLL1 and JAG1 was upregulated (*p* < 0.05, *p* < 0.01) (Fig. 2A - B′). In contrary, DLL4 was reduced in the testes of both experimental models (*p* < 0.01, *p* < 0.001) (Fig. 2C, C’).

**Fig. 3:**
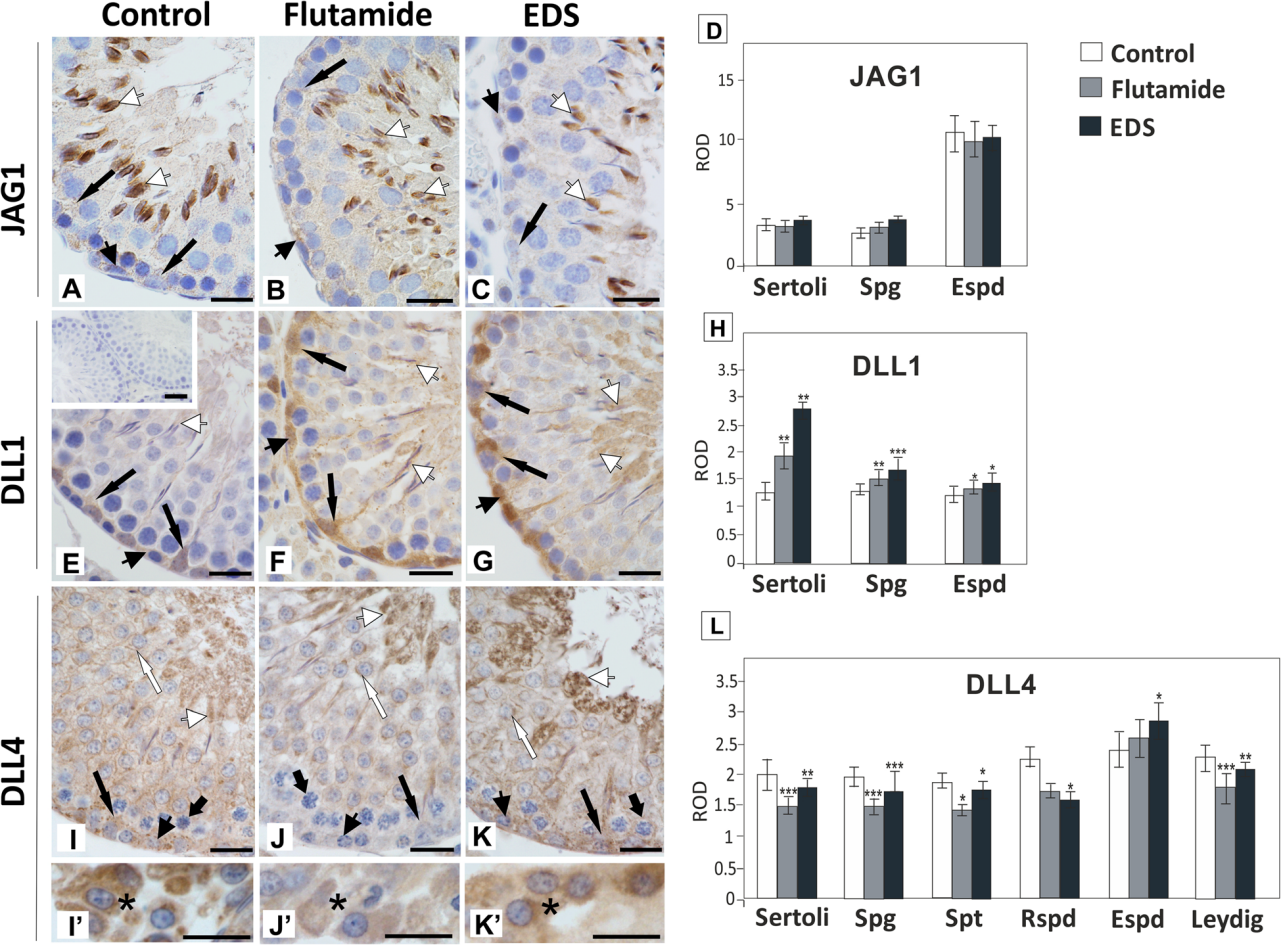
Effect of flutamide and EDS on JAG1, DLL1 and DLL4 immunoexpression in peripubertal rat testis. Representative micrographs are obtained from three independent analyses. Images A – C, E – G, and I - K represent seminiferous tubule sections, images I′ – K′ represent interstitial tissue. Negative control included section incubated with non-immune serum instead of the primary antibody (insert in E). Scale bar = 20 μm. Positive signals of the proteins are depicted by arrows. Sertoli cells – arrows, spermatogonia – arrowheads, pachytene spermatocytes – short arrows, round spermatids – white arrows, elongated spermatids – white arrowheads, Leydig cells – asterisks. Quantitative analysis of the intensity of immunocytochemical stainings expressed as relative optical density (ROD) of diaminobenzidine brown reaction product (D, H, L). The histograms are the quantitative representation after densitometry of data (mean ± SD) of three independent analyses. Significant differences from control values are denoted as *p < 0.05, **p < 0.01, and ***p < 0.001. Spg – spermatogonia, Spt – spermatocytes, Rspd – round spermatids, Espd – elongated spermatids

Three Notch pathway ligands JAG1, DLL1 and DLL4 were detected in seminiferous epithelium of pubertal rats (Fig. 3A - C, E - G, I - K), whereas in Leydig cells only DLL4 was found (Fig. 3I’- K′). JAG1 was localized predominantly in elongating spermatids, weak staining was also found in spermatogonia and Sertoli cells. An increase of JAG1 protein expression was not evident in immunohistochemical analysis (Fig. 3A - D). DLL1 was expressed in Sertoli cells, spermatogonia, and elongated spermatids. Immunohistochemistry followed by densitometry revealed highest DLL1 increase in Sertoli cells (*p* < 0.01) (Fig. 3E - H). DLL4 was the most ubiquitous ligand in seminiferous epithelium, being localized in Sertoli cells and germ cell throughout seminiferous epithelium (*p* < 0.05, *p* < 0.01, *p* < 0.001) (Fig. 3I). The most severe decrease of DLL4 signal was found in basal compartment of seminiferous epithelium of flutamide-treated rats (Fig. 3J). Notably, DLL4 signal was increased in elongated spermatids present in EDS-treated males (*p* < 0.05) (Fig. 3K, L).

**Fig. 4:**
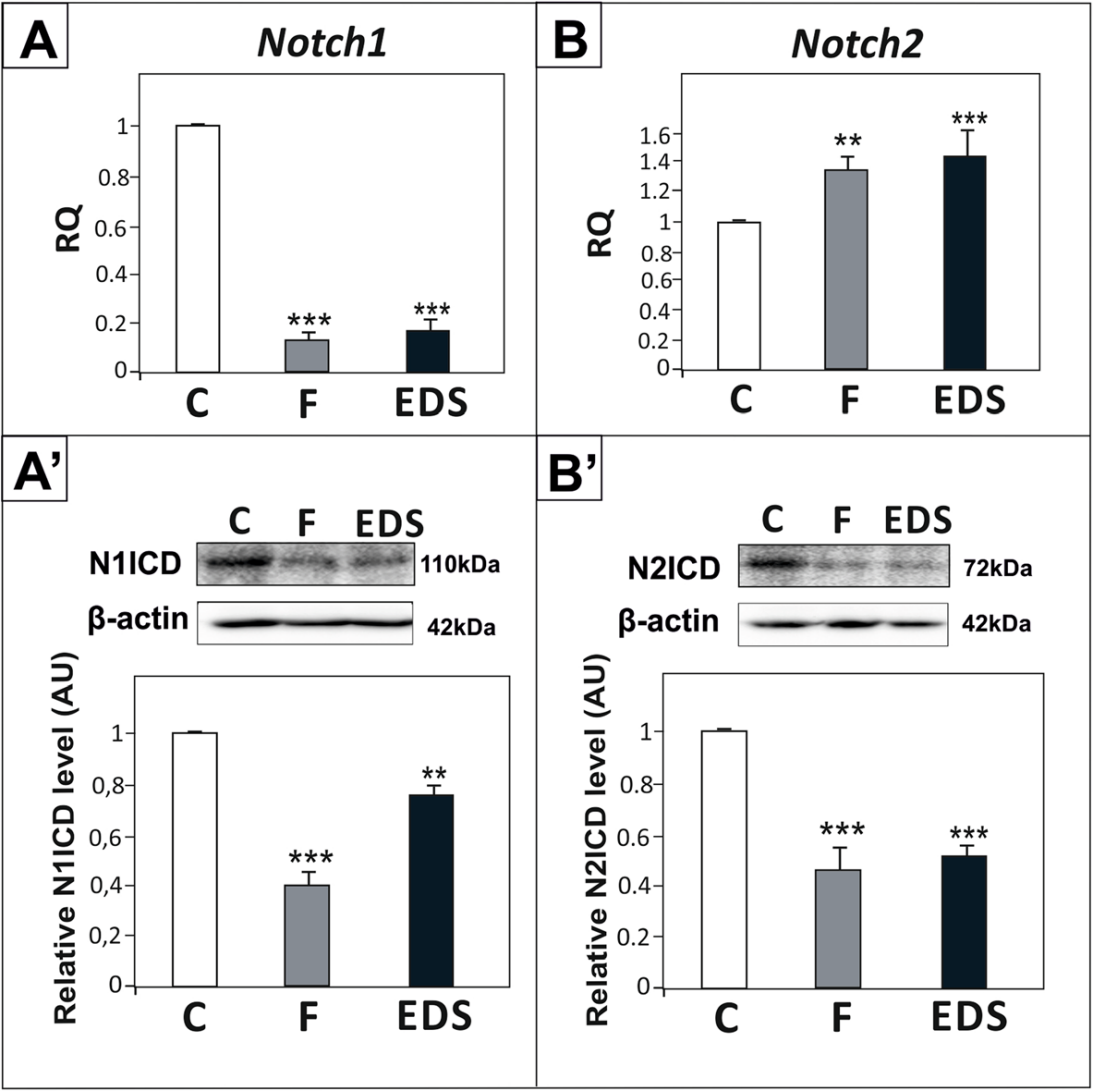
Effect of flutamide (F) and EDS on *Notch1* and *Notch2* expression in peripubertal rat testis. (A, B) Relative expression of *Notch1* and *Notch2* mRNAs was determined using real-time RT-PCR analysis. The histograms are the quantitative representation of data of three independent analyses (*n* = 6 each group). The expression values of the individual genes were normalized to the mean expression of the reference genes (*Rn18s*, *B2m* and *Actb*) as an internal control. Relative quantification (RQ) is expressed as mean ± SD. Significant differences from control values are denoted as **p < 0.01 and ***p < 0.001. (A’, B′) Relative protein levels of N1ICD and N2ICD were determined using western blot. The histograms are the quantitative representation after densitometry of data (mean ± SD) of three independent analyses (*n* = 6 each group). The relative level of studied protein was normalized against its corresponding actin data point. The protein levels within the control group were arbitrarily set as 1. Significant differences from control values are denoted as *p < 0.05, **p < 0.01, and ***p < 0.001

The expression of Notch1 receptor mRNA as well as the level of its active form N1ICD were significantly reduced in flutamide and EDS treated groups (*p* < 0.01, *p* < 0.001) (Fig. 4A, A’). In both experimental groups reduced N2ICD level was also detected, in spite of up-regulated *Notch2* mRNA expression (*p* < 0.01, *p* < 0.001) (Fig. 4B, B’).

**Fig. 5:**
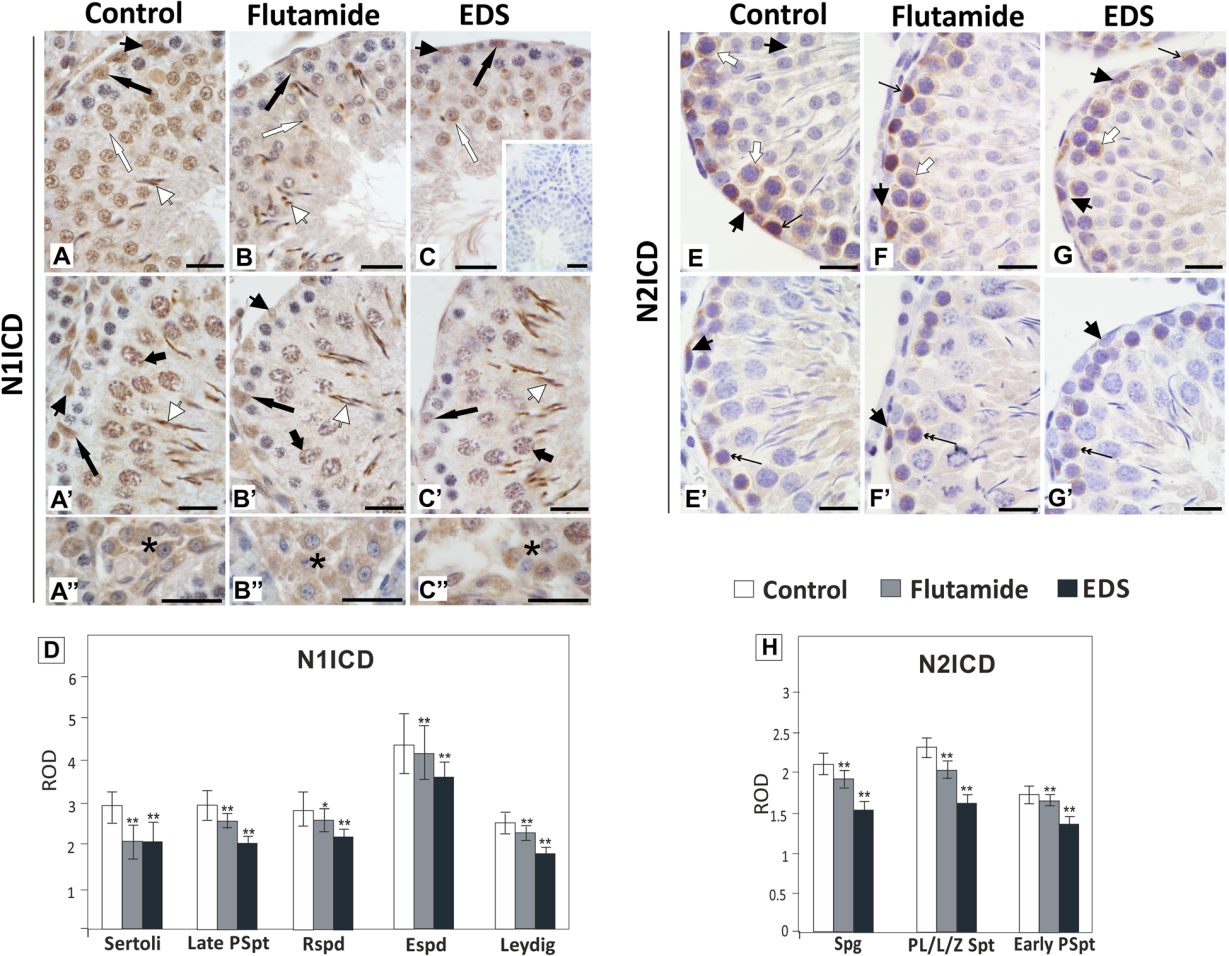
Effect of flutamide and EDS on N1ICD and N2ICD immunoexpression in peripubertal rat testis. Representative micrographs are obtained from three independent analyses. Images A – C and E – G represent early stages of seminiferous epithelium cycle, images A’ – C′ and E’ – G’ represent late stages of seminiferous epithelium cycle, images A” – C″ represent interstitial tissue. Negative control included section incubated with non-immune serum instead of the primary antibody (insert in C). Scale bar = 20 μm. Positive signals of the proteins are depicted by arrows. Sertoli cells – arrows, spermatogonia – arrowheads, preleptotene spermatocytes – open arrows, leptotene/zygotene spermatocytes - double arrows, early pachytene spermatocytes - short white arrows, late pachytene spermatocytes – short arrows, round spermatids – white arrows, elongated spermatids – white arrowheads, Leydig cells – asterisks. Quantitative analysis of the intensity of immunocytochemical stainings expressed as relative optical density (ROD) of diaminobenzidine brown reaction product (D, H). The histograms are the quantitative representation after densitometry of data (mean ± SD) of three independent analyses. Significant differences from control values are denoted as *p < 0.05, and **p < 0.01. Spg – spermatogonia, PL/L/Z Spt - preleptotene/leptotene/zygotene spermatocytes, Early PSpt - early pachytene spermatocytes, Late PSpt - late pachytene spermatocytes, Rspd – round spermatids, Espd – elongated spermatids

As demonstrated by immunohistochemistry, activated form of Notch1 receptor, N1ICD, was expressed in basal compartment of seminiferous epithelium in Sertoli cells and spermatogonia, then appeared in late pachytene spermatocytes and continued to be present in germ cells up to elongated spermatids (Fig. 5A, A’). N1ICD showed nuclear localization in the cells of seminiferous epithelium (Fig. 5A, A’), whereas in Leydig cells both nuclear and cytoplasmic signal was detected (Fig. 5A”). Apparent decrease of N1ICD immunoexpression was found in both seminiferous epithelium and Leydig cells of flutamide and EDS-treated rats (*p* < 0.05, *p* < 0.01) (Fig. 5B, B’, B″, C, C′, C″, D).

The highest immunoexpression of full-length Notch2 was observed in adluminal compartment of seminiferous epithelium and in the interstitial tissue (Additional Fig. 1), whereas the localization of N2ICD was restricted to early steps of germ cell differentiation, from spermatogonia up to early pachytene spermatocytes (Fig. 5E - G’). The most prominent decrease of N2IDC was observed in spermatogonia, preleptotene and leptotene/zygotene spermatocytes of EDS-treated males (*p* < 0.01) (Fig. 5H).

**Fig. 6:**
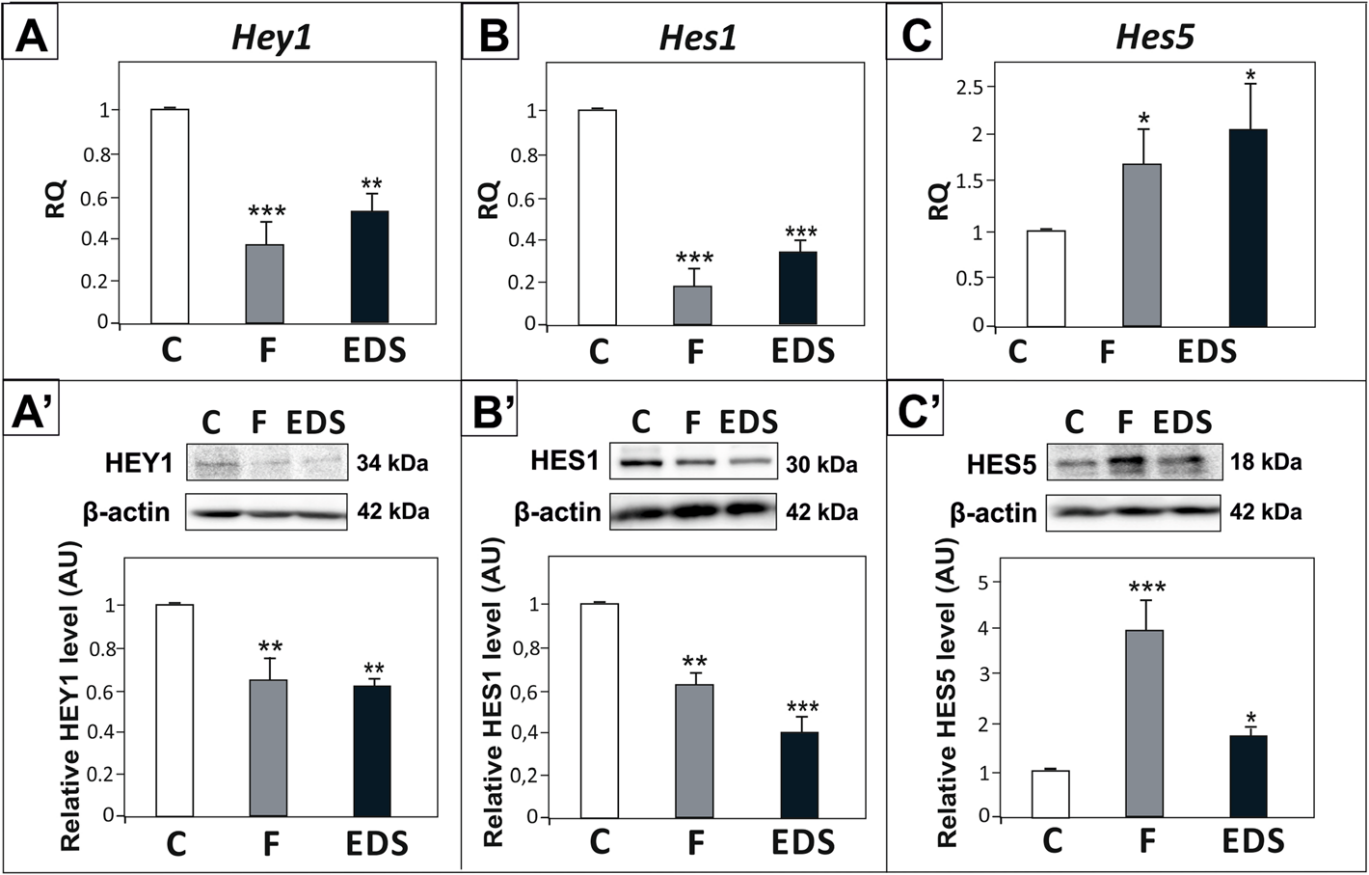
Effect of flutamide (F) and EDS on *Hey1*, *Hes1*, and *Hes5* expression in rat testis. (A - C) Relative expression of *Hey1, Hes1,* and *Hes5* mRNAs was determined using real-time RT-PCR analysis. The histograms are the quantitative representation of data of three independent analyses, each in triplicate (*n* = 6 each group). The expression values of the individual genes were normalized to the mean expression of the reference genes (*Rn18s*, *B2m* and *Actb*) as an internal control. Relative quantification (RQ) is expressed as mean ± SD. Significant differences from control values are denoted as **p < 0.01 and ***p < 0.001. (A’- C′) Relative protein expression of HEY1, HES1 and HES5 was determined using western blot. The histograms are the quantitative representation after densitometry of data (mean ± SD) of three independent analyses (*n* = 6 each group). The relative level of studied protein was normalized against its corresponding actin data point. The protein levels within the control group were arbitrarily set at 1. Significant differences from control values are denoted as *p < 0.05, **p < 0.01, and ***p < 0.001

Attenuation of testosterone signaling had differential effects on the expression of the effector genes. Significant reduction of *Hey*1 and *Hes1* mRNA and protein levels was found following androgen withdrawal (*p* < 0.01, *p* < 0.001) (Fig. 6A – B′). In contrast, *Hes5* mRNA and protein expressions were upregulated (*p* < 0.05, *p* < 0.001) (Fig. 6C, C’).

**Fig. 7:**
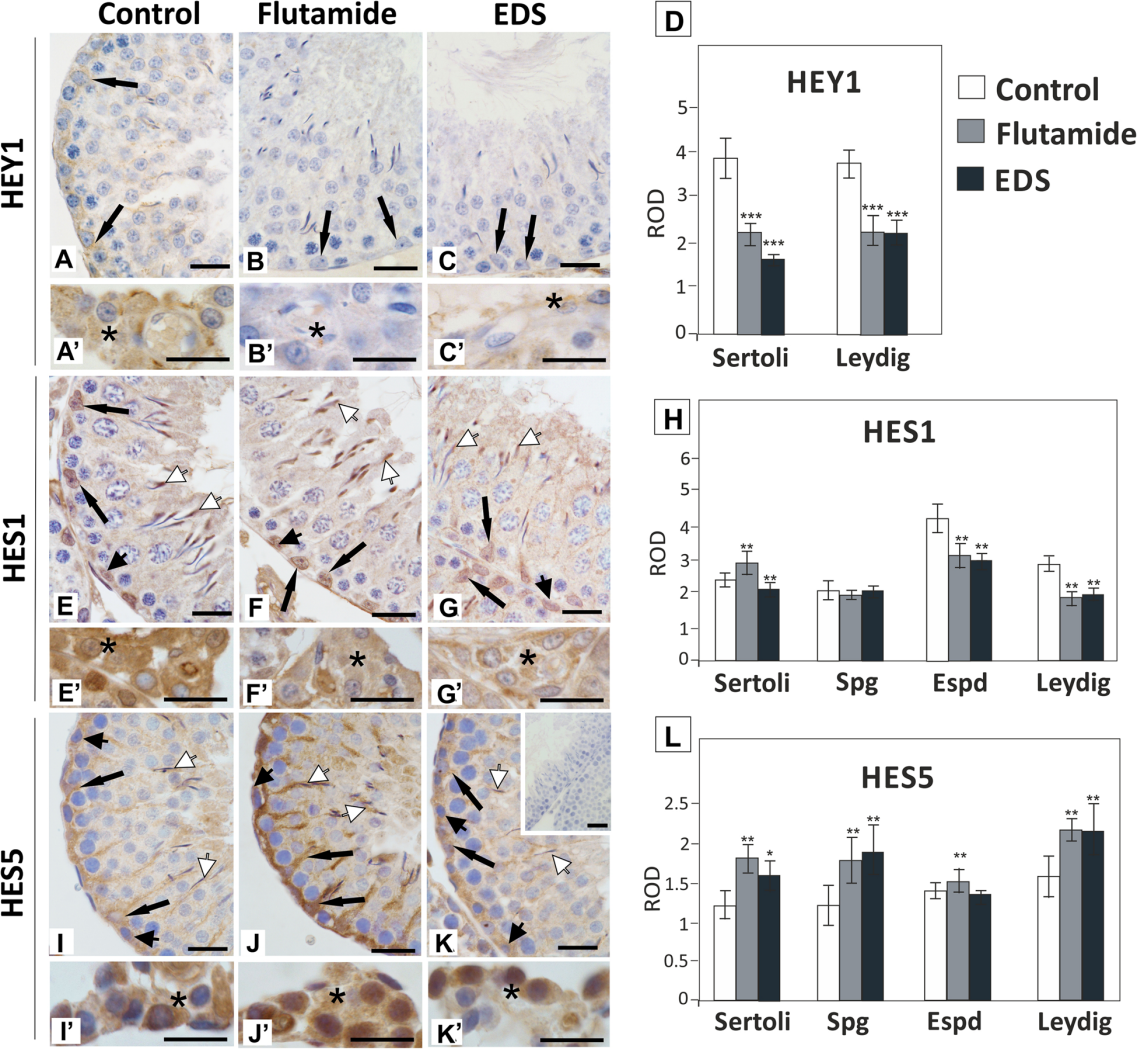
Effect of flutamide and EDS on HEY1, HES1 and HES5 immunoexpression in peripubertal rat testis. Representative micrographs are obtained from three independent analyses. Images A – C, E – G, and I - K represent seminiferous tubule sections, images A’ – C′, E’ – G’, and I′ – K′ represent interstitial tissue. Negative control included section incubated with non-immune serum instead of the primary antibody (insert in K). Scale bar = 20 μm. Positive signals of the proteins are depicted by arrows. Sertoli cells – arrows, spermatogonia – arrowheads, elongated spermatids – white arrowheads, Leydig cells – asterisks. Quantitative analysis of the intensity of immunocytochemical stainings expressed as relative optical density (ROD) of diaminobenzidine brown reaction product (D, H, L). The histograms are the quantitative representation after densitometry of data (mean ± SD) of three independent analyses. Significant differences from control values are denoted as *p < 0.05, **p < 0.01, and ***p < 0.001. Spg – spermatogonia, Espd – elongated spermatids

HEY1 was restricted to Sertoli cells and Leydig cells, where it produced very weak cytoplasmic signal (Fig. 7A, A’). In both flutamide and EDS-treated rats HEY1 signal almost completely disappeared; significant reduction of immunostaining intensity was confirmed with densitometry (*p* < 0.01) (Fig. 7B - D). In seminiferous epithelium HES1 exhibited exclusively nuclear localization and was detected in Sertoli cells, spermatogonia, and elongated spermatids (Fig. 7E). Nuclear and cytoplasmic staining was found in Leydig cells (Fig. 7E’). The reduction of HES1 immunoexpression was detected in elongated spermatids in both experimental groups, and in Sertoli cells of EDS-treated males (Fig. 7F, F’, G, G’, H). In rats exposed to flutamide a slight increase of immunostaining was found in Sertoli cells (*p* < 0.001) (Fig. 7F, H). HES5 was detected in Sertoli cells, spermatogonia, elongated spermatids and Leydig cells; the most noticeable increase of HES5 immunoreactivity was found in basal compartment of seminiferous epithelium of flutamide-treated rats (*p* < 0.01) (Fig. 7I - L).

## Discussion

In the present study we found that androgen signaling in peripubertal males controls the expression of Notch pathway ligands and activity of Notch signaling in the testis. To reveal this interaction, we reduced androgen signaling in rats by either blocking the AR or testosterone production. It should be noted that elimination of Leydig cells with EDS not only reduces the level of testosterone, but also affects production of other Leydig cell-derived factors. However, in both models used in our study similar changes in the expression of Notch pathway components in seminiferous epithelium were found, which indicates that disruption of androgen action is the main reason of observed alterations. Furthermore, the changes were usually more evident when the AR was blocked. This observation suggests that classical pathway of androgen action via AR is an important mechanism responsible for the regulation of Notch signaling in pubertal testis by androgens. Interaction between AR signaling and Notch pathway was earlier found in human endometrial cell lines MFE-296 and AN3CA; overexpression of the AR resulted in increased expression of Notch1 and Hes1, which was attenuated by co-transfection with AR siRNA [35].

Analysis of the expression of Notch ligands following androgen signaling disruption revealed differential responses of particular ligands. It is likely that in the testis, similarly as in other tissues, the balance between Notch ligands is important for undisturbed functioning [36]. Of note, different ligands expressed in one tissue often have opposing activities [37, 38]. Therefore, the maintenance of balanced expression and action of the ligands may result from their diverse (antagonistic) regulation by a common factor, as it was demonstrated in breast cancer cells [39].

DLL4 decreased significantly following androgen signaling withdrawal, which correlated with decreased level of the active form of Notch1 receptor in Sertoli cells. Based on IHC results, DLL4 decrease in basal compartment of seminiferous epithelium seems to be strongly dependent on AR blockade. Since the AR is detected only in somatic testicular cells, changes in ligand expression in germ cells may result from indirect effects mediated likely via Sertoli cells. In case of EDS-induced testosterone withdrawal, decrease of *Dll4* mRNA and protein level might be ascribed predominantly to the loss of Leydig cells, however reduced immunoexpression in seminiferous epithelium was also observed.

In contrast to DLL4, DLL1 and JAG1 appeared to be negatively regulated by androgens since both flutamide and EDS exposure resulted in up-regulation of their expressions. It should be mentioned that increased expression of Notch ligands may exert either stimulatory or inhibitory effect on Notch pathway activation. In case of the expression of both ligand and receptor within the same cell, response called cis-inhibition is observed [40]. In this study DLL1 expression was enhanced predominantly in Sertoli cells in which the activation of Notch1 receptor significantly decreased, which suggest the possibility of above-mention effect.

The expression of JAG1 was found mainly in elongating spermatids of pubertal rats. In our study both spermatogonia and Sertoli cells exhibited only scarce signal, indicating low level of this protein. Such localization was in contrast to those observed previously in adult rat, where the highest expression of this ligand was detected in spermatogonia [41, 42]. In adult mouse, however the localization of JAG1, performed with the same technique as used in the present study was in agreement with our findings [9]. Although both qRT-PCR and western blot analyses revealed increased JAG1 expression after flutamide, IHC followed by densitometry did not demonstrate significant changes of signal intensity. The limitation of this study lies in semi-quantitative nature of densitometry analysis of immunohistochemical staining. The relationship between the concentration of a DAB reaction product and its absorbance is not linear and DAB-based immunohistochemistry has limited dynamic range [43]. As such, this approach has limited resolution in measuring modest differences and some minor changes in protein expressions between experimental groups might be undetectable with this method.

Since JAG1 was localized mostly to elongating spermatids, where it produced strong signal, it is likely that further increase of the signal was not detectable using densitometric approach. On the other hand, attempts to reduce signal intensity in spermatids resulted in concomitant complete loss of the signal in Sertoli cells and spermatogonia. Thus, it is likely that overall increase of JAG1 protein expression came from elongated spermatids. Nevertheless, further studies are needed in order to clearly identify cells in which JAG1 expression is androgen regulated.

Altogether, the expression of Notch ligands was deregulated after depletion of testosterone signaling. It is likely that these changes led directly to decreased activation of Notch1 and Notch2 receptors. However, the reduction in N1ICD level maybe a consequence of downregulation of *Notch1* mRNA expression as well, because we found attenuation of both *Notch1* mRNA and cleaved Notch1 protein expression.

Decreased expression of N1ICD in seminiferous epithelium was accompanied by the reduction of HEY1 expression in Sertoli cells after EDS. Slight increase of HES1 found in Sertoli cells of flutamide-treated rats may suggest that regulation of *Hes1* expression in these cells is controlled via AR-independent androgen signaling. Such non-classical androgen pathways have been recently revealed [44], but their possible role in the control of *Hes1* requires further research.

To date the significance of Notch1/HES1/HEY1 pathway in the testis was precisely determined only in Sertoli cells. It was established that HES1 and HEY1 inhibit the production of glial cell line-derived neurotrophic factor (GDNF) and expression of retinoid acid metabolizing enzyme CYP26B1 by Sertoli cells [42, 45]. The results by Murta et al. [13] suggested also a regulatory role of Notch signaling in spermatid elongation and maintenance of the elongated spermatid anchoring system. Notably, according to earlier findings elongating spermatids development is androgen-dependent [46, 47].

It is worth mentioning that in mouse HES1 was detected in spermatogonia of immature testis. In adult mice the expression in spermatogonia decreased and HES1 was detected mainly in Sertoli cells [9, 15, 48]. In pubertal rats we found the expression of this protein in both spermatogonia and Sertoli cells. However, in contrary to the observations made in mice, in rat we detected clear HES1 signal also in elongated spermatids. This may suggest either species-dependent localization or expression specific for the first wave of spermatogenesis. Inhibition of androgen signaling or production led to reduced immunoexpression of HES1 in elongated spermatids. In the light of data presented herein, the interaction between androgen and Notch signaling may be important during spermiogenesis and is worth further in-depth investigation.

In agreement with the results of the present study an environmental endocrine disruptor possessing anti-androgenic activity, di-(2-ethylhexyl) phthalate, reduced Notch1 and HES1 expression in adult rat testis [49]. Our previous study demonstrated increased activation of Notch1/HEY1 pathway and decrease of HES5 following dibutyl phthalate exposure of adult rat testis *in vitro* [31]. The reason of this discrepancy is unclear; it cannot be however excluded that the latter effect was due to some other mechanisms of dibutyl phthalate action e.g. estrogenic or AhR-dependent [50–52].

To our knowledge there are no reports on N2ICD localization or function in rodent testes. Full-length Notch2 receptor appears in peripubertal mouse testis in germ cells entering meiosis and in adult rats is present in pachytene spermatocytes, spermatids and Leydig cells [9, 19]. Similarly, our results demonstrated the highest immunoexpression of Notch2 in adluminal compartment of seminiferous epithelium and in the interstitial tissue of peripubertal rats. Surprisingly, this is in contrast to the localization of activated form of Notch2; we detected N2ICD predominantly in pre-meiotic germ cells and no signal was detected in Leydig cells. These observations suggest a possible role of Notch2-dependent signaling in early steps of germ cell differentiation. Androgen signaling deprivation resulted in decreased level of N2ICD in testes of experimental rats. However, the expression of Notch2 receptor mRNA was up-regulated. This may indicate that androgen signaling potentiates Notch2 receptor activation, but simultaneously is involved in the maintenance of proper level of Notch2 receptor expression in seminiferous epithelium of pubertal males.

N2ICD co-localized with Notch effector proteins only in spermatogonia, where HES1 and HES5 were detected. However, we did not observe significant effect of androgen signaling inhibition on HES1 immunoexpression in spermatogonia, whereas HES5 increased in these cells. Thus neither of the targets investigated herein seems to mediate Notch2 signaling in spermatogonia and the downstream pathway for Notch2 in seminiferous epithelium remains to be investigated. Nevertheless, the presence of N1ICD and N2ICD and Notch effector genes in spermatogonia strongly suggest that Notch pathway is involved in the control of these cells. In addition, Okada et al. [15] showed the expression of cleaved form of Notch3 receptor (N3ICD) and HES1 in prepubertal and adult mouse spermatogonia. Altogether, Notch signaling is clearly activated in spermatogonia via different Notch receptors, but whether each of them play specific role or they are functionally redundant remains to be explained.

Noteworthy, despite reduced activation of Notch receptors, the expression of HES5 was increased, most markedly in flutamide-treated males. This may suggest that in peripubertal testis androgens affect HES5 expression through AR-mediated, but Notch-independent mechanism. Such possibility was evidenced earlier in prostate cancer cells where another member of HES family, HES6, was regulated by androgens without involvement of Notch signaling [53]. Recently the role of AR in control of *Hes5* expression was also found in spinal and bulbar muscular atrophy model [54].

Although this study has focused on seminiferous epithelium, we found that the expression of Notch pathway components was affected in Leydig cells following flutamide administration. In this model DLL4, N1ICD, HES1 and HEY1 were clearly reduced, which indicates that AR activity is required for maintenance of proper levels of these proteins in Leydig cells. Nevertheless, the role of Notch pathway in rat Leydig cell physiology during puberty is not defined and needs further investigation. The only protein negatively regulated by AR in Leydig cells was HES5. This effect, similarly as in seminiferous epithelium, may be explained by direct control of *Hes5* expression by AR signaling.

## Conclusion

Our results demonstrated for the first time that androgens and the AR may be considered as factors regulating Notch pathway activity and the expression of *Hes* and *Hey* genes in rat seminiferous epithelium during pubertal development. In our recent study we found that DLL1 and JAG1 ligands and Notch1 receptor activity are involved in the regulation of androgen receptors expression and action in Sertoli cells *in vitro* [55]. Altogether, these observations indicate the interrelationship between androgen and Notch pathways in seminiferous epithelium. Further studies should focus on functional significance of androgen-Notch signaling cross-talk in spermatogenesis.

## Supplementary information

**Additional file 1.** Immunoexpression of full-length Notch2 protein in rat testis. Scale bar = 20 μm. Cells showing highest signal intensity are depicted by arrows: late pachytene spermatocytes – short arrows, round spermatids – white arrows, elongated spermatids – white arrowheads, Leydig cells – asterisks.

## Data Availability

The datasets analyzed during the current study are available from the corresponding author on reasonable request.

## Abbreviations

*Actb*: β-actin
ADAM: A disintegrin and metalloproteinase
AhR: Aryl hydrocarbon receptor
AR: Androgen receptor
*B2m*: β-2-microglobulin
CYP26B1: Cytochrome P450 26B1
*Dll1*/DLL1: Delta-like 1
Dll4/DLL4: Delta-like 4
EDS: Ethanedimethane sulphonate
GDNF: Glial cell line-derived neurotrophic factor
*Hes1*/HES1: Hairy/enhancer of split 1
*Hes5*/HES5: Hairy/enhancer of split 5
*Hey1*/HEY1: Hes-related with YRPW motif 1
Jag1/JAG1: Jagged 1
LHR: Luteinizing hormone receptor
MAML1: Mastermind-like 1
N1ICD: Notch1 intracellular domain
N2ICD: Notch2 intracellular domain
NICD: Notch intracellular domain
PD: Postnatal day
RBP-J: Recombination signal binding protein
*Rn18s*: 18S ribosomal RNA
