# Supplementary figures and images for: Disruption of androgen signaling during puberty affects Notch pathway in rat seminiferous epithelium
Source: Reprod Biol Endocrinol. 2020 Apr 16;18:30. doi: 10.1186/s12958-020-00582-3 (PMC7161021; doi:10.1186/s12958-020-00582-3)

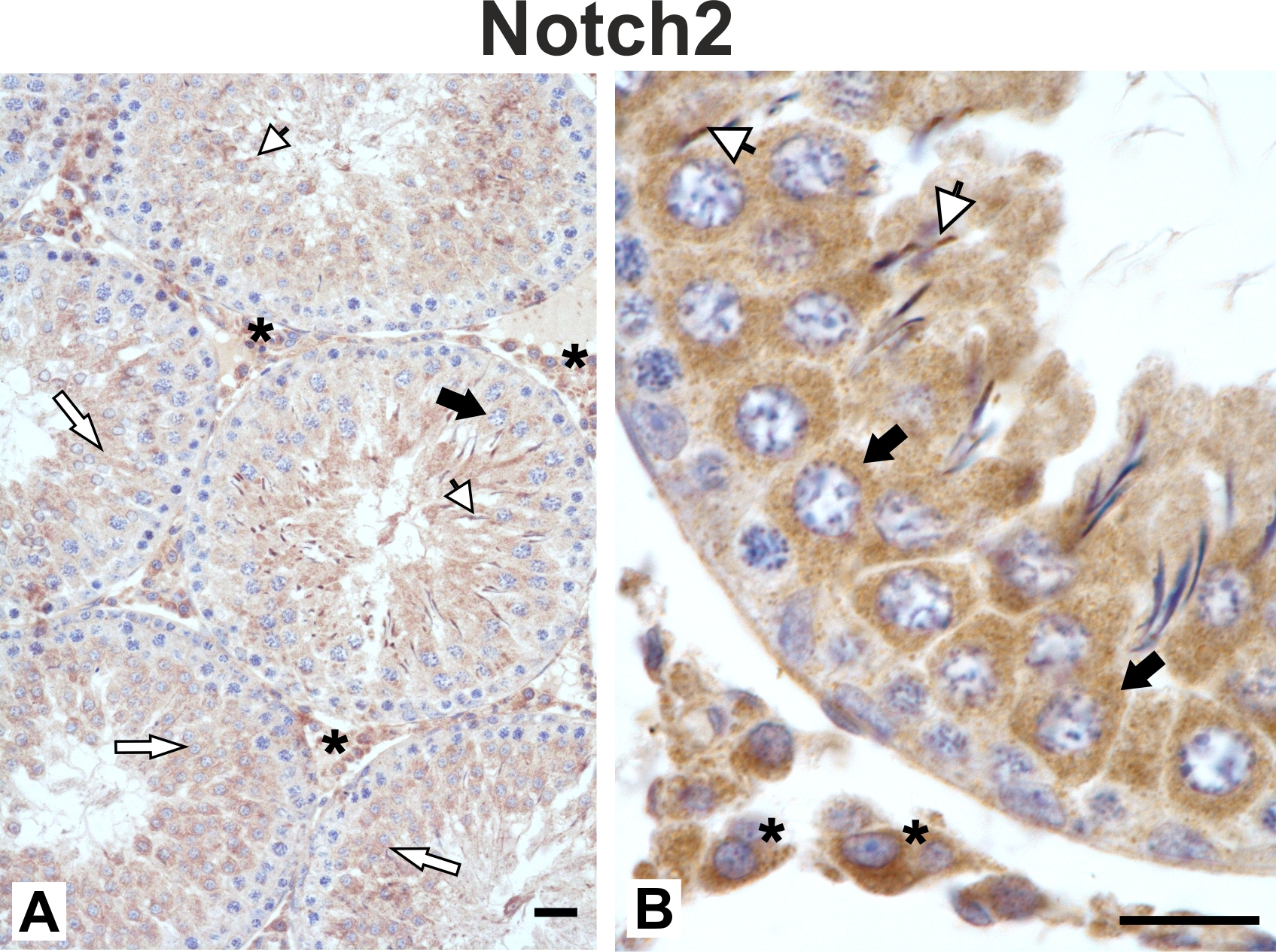

Supplement: Supplementary file 1 — Additional file 1. Immunoexpression of full-length Notch2 protein in rat testis. Scale bar = 20 μm. Cells showing highest signal intensity are depicted by arrows: late pachytene spermatocytes – short arrows, round spermatids – white arrows, elongated spermatids – white arrowheads, Leydig cells – asterisks. [file 12958_2020_582_MOESM1_ESM.jpg]
